# Acute Monocytic Leukemia Masquerading Behçet's Disease-Like Illness at Onset in an Elderly Female

**DOI:** 10.1155/2016/4231276

**Published:** 2016-08-16

**Authors:** Shigeru Koba, Toshio Sekioka, Sorou Takeda, Aya Miyagawa-Hayashino, Keisuke Nishimura, Shinsaku Imashuku

**Affiliations:** ^1^Department of Internal Medicine, Uji-Tokushukai Medical Center, Uji 611-0042, Japan; ^2^Department of Diagnostic Pathology, Kyoto University Hospital, Kyoto 606-8507, Japan; ^3^Division of Pathology, Uji-Tokushukai Medical Center, Uji 611-0042, Japan; ^4^Department of Laboratory Medicine, Uji-Tokushukai Medical Center, Uji 611-0042, Japan

## Abstract

A previously healthy 74-year-old Japanese female was hospitalized with fever and high C-reactive protein. She developed palatal herpangina-like aphthous ulcers, localized intestinal wall thickening, terminal ileum ulcers, and an erythematous acneiform rash; thus Behçet's disease-like illness was suspected. Significant peripheral blood acute monocytosis developed during her hospitalization and acute monocytic leukemia (FAB M5b) with normal karyotype was diagnosed. By immunostaining, the infiltrating cells in the skin and the terminal ileum were identified as monocytic leukemic cells. This case exhibited a unique initial presentation of Behçet's disease-like illness associated with acute monocytic leukemia.

## 1. Introduction

Autoimmune diseases like Behçet's disease (BD) develop in as many as 10% of patients with myelodysplastic syndromes (MDS) or myeloproliferative neoplasms (MPN) [1]. In particular, a link between BD or BD-like illness and chronic myelomonocytic leukemia (CMML) with a trisomy 8 chromosome abnormality has been well recognized [2–4]. However, the association of BD or BD-like illness and acute myeloid leukemia has rarely been reported [5]. In patients with CMML showing cutaneous lesions as a sign of BD or BD-like illness, skin tissues were shown to contain abnormal monocytes, that is, leukemia cutis [6–8]. We report here an elderly Japanese female who initially presented with BD-like illness including cutaneous lesions and thereafter was diagnosed as having acute monocytic leukemia (AMoL; FAB M5b). In this case, we identified leukemic cells in the tissues of skin and ileum.

## 2. Case Report

**Figure 1:**
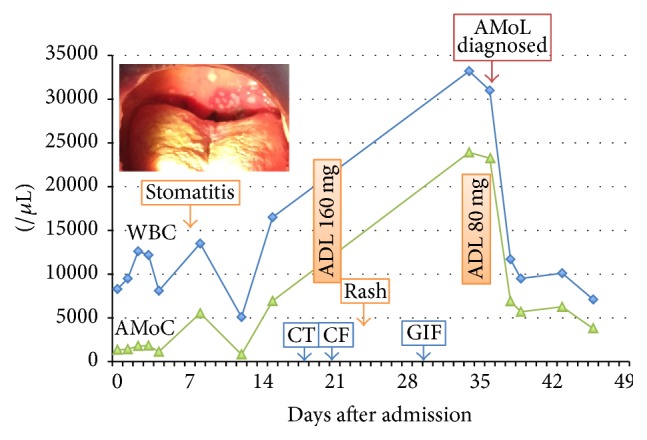
Clinical course of the patient: the patient first developed aphthous stomatitis (inserted photo), followed by intestinal BD-like lesions detected by CF and then skin rash. Significant monocytosis triggered by urinary tract infection was noted at the 5th week of admission and the eventual diagnosis of AMoL was made. WBC: white cell counts, AMoC: absolute monocyte counts, ADL: adalimumab, CT: computerized tomography, CF: colonofiberscopy, GIF = gastrointestinal fiberscopy.

**Figure 2:**
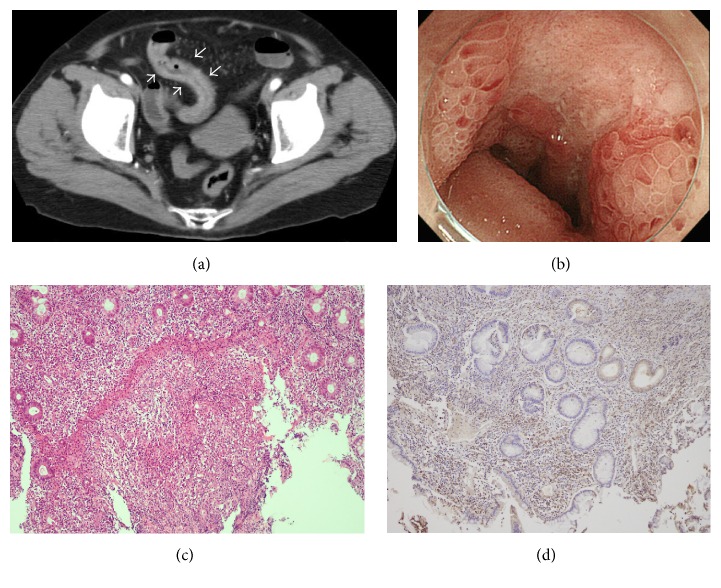
Intestinal findings. Abdominal CT scan showed localized thickening of the intestinal wall (arrows) (a). Endoscopic study revealed multiple ovoid punched-out ulcers at the terminal ileum (b). HE staining of a biopsy of the terminal ileum showed the presence of ulcer-associated granulation tissues with vascularization and infiltration of cells (original magnification ×100) (c), which were strongly positive for lysozyme (d) (original magnification ×100). Positive stains for CD13, CD14, and CD33 are not shown.

**Figure 3:**
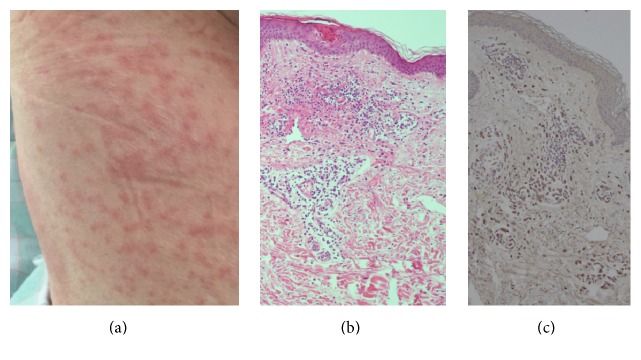
Findings of cutaneous lesions: photo of the rash on the right thigh (a). The biopsy shows the dense perivascular and periadnexal infiltration of mononuclear cells in the dermis extending into the subcutaneous tissue (b) (HE staining, original magnification ×100). The mononuclear cells were strongly lysozyme-positive (c) (original magnification ×100). Positive stains for CD13, CD14, and CD33 are not shown.

**Figure 4:**
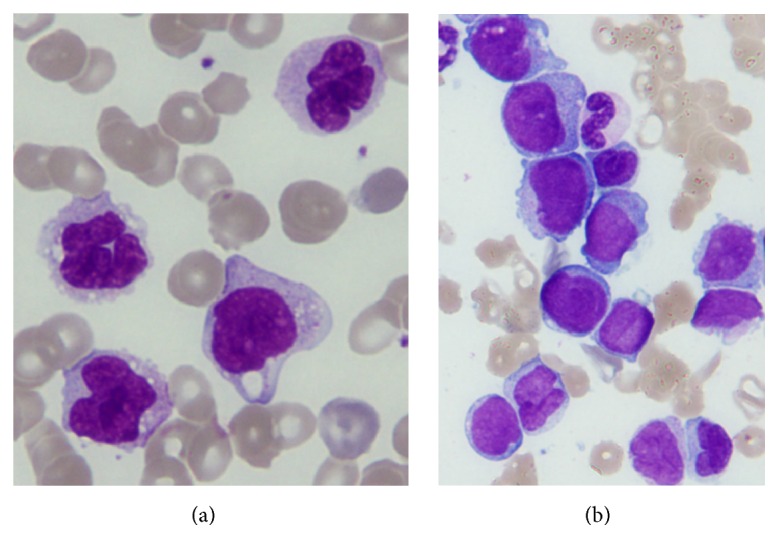
May-Grünwald-Giemsa stained smears of peripheral blood and bone marrow show mature monocytes and promonocytoid cells with folded nuclei in the peripheral blood (a) and immature blast and promonocytoid cells in the bone marrow (b) (original magnification ×1,000).

A previously healthy 74-year-old Japanese female was hospitalized with unknown fever and high C-reactive protein (CRP) values in December 2015. On admission, she was 155 cm in height and 59.2 kg in weight and had temperature of 37.6°C, blood pressure of 118/64 mmHg, pulse rate of 71/min, respiration rate of 18/min, and SpO_2_ of 94% at room temperature. Physical examination revealed no specific findings. After admission, she developed herpangina-like aphthous ulcers at the palate, when significant monocytosis (absolute monocyte counts >5,000/*μ*L) was first noted; however, monocytosis fluctuated (Figure 1). Thereafter, a CT scan of the abdomen showed localized thickening of the intestinal wall (Figure 2(a)) and colonofiberscopy (CF) revealed the presence of multiple ovoid punched-out ulcers at the terminal ileum and aphthous lesions at the ascending colon (Figure 2(b)). In addition, the patient also developed multiple erythematous rashes on her right thigh (Figure 3(a)). Given the diagnostic criteria of BD [9], the rash was not typical erythema nodosum but was thought rather to be acneiform eruption. Ophthalmological studies showed no evidence of BD signs. Taken together, BD was suspected in this patient from fulfilling 2 major and 1 minor clinical features required in the diagnosis of BD [9]. Thereafter, from the second week of admission, the patient again developed a significant monocytosis (from 15% to >70% in PB, with absolute monocyte counts to a maximum of 23,900/*μ*L) associated with urinary infection at the 5th week of admission (Figure 1). These findings with subsequent bone marrow study confirmed a diagnosis of AMoL (Figures 4(a) and 4(b)).

Blood results at the 5th week of admission, when AMoL was diagnosed, were as follows: white blood cells (33,200/*μ*L) containing 15% blasts, 21% promonocytes, 19% mature monocytes, 1% myelocytes, 10% neutrophils, and 33% lymphocytes (Figure 4(a)). Flow cytometric data of a major cell population in PB were as follows: CD13+ (86.4%), CD14+ (80.0%), CD33+ (97.4%), CD34+ (0.8%), CD41+ (44.8%), CD56+ (32.4%), and HLA-DR+ (97.0%). Mature lymphocytes in PB consisted of mostly T cells (approximately 90%), whose markers were CD4+ (59.2%) and CD8+ (27.4%), respectively. By contrast, the hypercellular BM consisted of 33.2% blasts, 15.0% promonocytes, 33.6% mature monocytes, 6.8% granulocytes, 1.4% lymphocytes, 2.0% plasma cells, and 8.0% erythroblasts with an M/E ratio of 0.85 (Figure 4(b)). No significant abnormalities indicating MDS were noted. Flow cytometric results of the major cell population in BM were as follows: CD13+ (38.3%), CD14+ (26.0%), CD33+ (97.5%), CD41+ (45.5%), CD34+ (2.3%), CD56+ (38.0%), and HLADR+ (80.9%). Mature lymphocytes in BM were mostly T cells (approximately 90%) with a CD4/CD8 ratio of 0.54. Myeloperoxidase positive cells accounted for 10% of the mononuclear cells present in both the PB and BM, while alpha-naphthyl butyrate esterase (inhibited by sodium fluoride) positive cells comprised more than 60% of cells in both the PB and BM. These findings were compatible with the diagnostic criteria of AMoL (FAB M5b) [10]. In addition, the karyotypes of PB and BM cells were both 46, XX [20/20]. The other blood chemistry was uneventful except for high levels of CRP (15.75 mg/dL), with aspartate aminotransferase 13 U/L, alanine aminotransferase 14 U/L, lactate dehydrogenase 205 U/L, total bilirubin 0.76 mg/dL, total protein 6.9 g/dL, albumin 2.9 g/dL, blood urea nitrogen 23.4 mg/dL, creatinine 1.61 mg/dL, sodium 135 mmol/L, potassium 3.6 mmol/L, chlorine 97 mmol/L, and calcium 8.0 mg/dL.

Since the diagnosis of AMoL (FAB M5b) was confirmed, the biopsied tissues showing BD-like illness were reevaluated with immunostaining except for stomatitis lesions and were proved to have leukemic cell infiltrations. The intestinal ulcer-associated granulation tissues with vascularization and infiltration of cells were positive for lysozyme, CD13, CD14, and CD33 (Figures 2(c) and 2(d)). Also the histopathology of skin rash showed that the infiltrating cells were positive for lysozyme, CD13, CD14, and CD33 (Figures 3(b) and 3(c)). These findings confirmed that the lesions primarily thought due to BD-like illness were in fact AMoL-related. In addition, HLA-typing, performed to ascertain whether the patient had BD-related HLA types, detected the presence of A2/A24 and B52/B55 but not the BD-related HLA-B51 or B5 alleles [11, 12]. As shown in Figure 1, the patient was initially treated with adalimumab for BD-like illness, following the successful report for a case of intestinal BD with trisomy 8 MDS by Kimura et al. [13]; however, after AMoL was confirmed, she was transferred to another hospital for intensive chemotherapy as a very high-risk patient.

## 3. Discussion

This case is not a classic autoimmune case of BD, nor is AMoL that developed during the treatment for BD. AMoL masquerading BD-like illness acutely developed. We had no evidence that this case progressed from CMML, because blood count records for the past 5 years prior to admission showed no increase of monocytes (which remained <10%) in the PB. Clinical features of BD, aphthous stomatitis, skin lesions, and ulcers in the terminal ileum were present, but genital ulcers, vascular, or ocular involvements were absent; thus, it was considered to be an incomplete BD or BD-like illness. The HLA-B51 allele was also absent. To date, the association of BD or BD-like illness with MDS has been well characterized, particularly in those patients with CMML [2–4, 14–16], but rarely characterized in patients with acute myeloid leukemia [5]. The precise causes developing BD or BD-like illness in cases of MDS/CMML are unknown [17].

In immunostaining studies in a classic autoimmune case of BD, Yamana et al. [18] showed that the lymphocytes infiltrating the terminal ileum were high CD4 (Leu 3a)^+^ cells and low CD8 (Leu 2a)^+^ cells. On the other hand, Tada et al. [14] previously reviewed the clinical characteristics of MDS-associated BD or BD-like illness in Japan but did not mention the characteristics of infiltrating cells in the tissues. In CMML cases, leukemic cells were reported to infiltrate cutaneous tissues as leukemia cutis [6–8]. We confirmed that both the skin rash and ileal tissues were infiltrated with monocytic leukemic cells in the case presented here. It remains unknown however how these findings explain the development of BD or BD-like illness in a case of AMoL. In a classic autoimmune case of BD cytokine-producing dysfunctional T cells play a major role [19]. In our case, infiltrating monocytic leukemic cells or T cells responding to monocytic leukemic cells or both might have played a similar role by producing various cytokines. As another interesting issue, a literature survey indicates a correlation of BD or BD-like illness and MDS/CMML with the presence of chromosomal trisomy 8 [2–4, 15, 16]. The present case, in contrast, had no trisomy 8.

In summary, although rare, caution must be exercised if BD or BD-like illness is associated with any hematological diseases, such as MDS/CMML, or rarely AMoL. We presented here the unique clinical course of an elderly patient whose disease initiated with the clinical features mimicking BD and was followed by the diagnosis of AMoL (FAB M5b) with normal karyotype.
